# The impact of tumor metabolic activity assessed by ^18^F-FET amino acid PET imaging in particle radiotherapy of high-grade glioma patients

**DOI:** 10.3389/fonc.2022.901390

**Published:** 2022-09-20

**Authors:** Maria Waltenberger, Jennifer Furkel, Manuel Röhrich, Patrick Salome, Charlotte Debus, Bouchra Tawk, Aoife Ward Gahlawat, Andreas Kudak, Matthias Dostal, Ute Wirkner, Christian Schwager, Christel Herold-Mende, Stephanie E. Combs, Laila König, Jürgen Debus, Uwe Haberkorn, Amir Abdollahi, Maximilian Knoll

**Affiliations:** ^1^ Department of Radiation Oncology, University Hospital of Heidelberg, Heidelberg, Germany; ^2^ Clinical Cooperation Unit Radiation Oncology, German Cancer Research Center (DKFZ), Heidelberg, Germany; ^3^ Heidelberg Institute for Radiation Oncology (HIRO), University Hospital of Heidelberg, Heidelberg, Germany; ^4^ Translational Radiation Oncology, German Cancer Consortium (DKTK), National Center for Tumor Diseases (NCT), German Cancer Research Center (DKFZ), Heidelberg, Germany; ^5^ Department of Radiation Oncology, Klinikum rechts der Isar, Technical University of Munich (TUM), Munich, Germany; ^6^ Department of Nuclear Medicine, University Hospital Heidelberg, Heidelberg, Germany; ^7^ Steinbuch Centre for Computing (SCC), Karlsruhe Institute of Technology (KIT), Eggenstein-Leopoldshafen, Germany; ^8^ Department of Experimental Neurosurgery, University Hospital Heidelberg, Heidelberg, Germany; ^9^ German Cancer Consortium (DKTK), Partner Site Munich, Munich, Germany; ^10^ Institute of Radiation Medicine (IRM), Helmholtz Zentrum Munich, Munich, Germany

**Keywords:** particle therapy, 18F-FET-PET, liquid biopsy, whole blood transcriptome, conformity index (CI), high grade glioma (HGG)

## Abstract

**Background:**

Selective uptake of (18)F-fluoro-ethyl-tyrosine (^18^F-FET) is used in high-grade glioma (HGG) to assess tumor metabolic activity *via* positron emission tomography (PET). We aim to investigate its value for target volume definition, as a prognosticator, and associations with whole-blood transcriptome liquid biopsy (WBT lbx) for which we recently reported feasibility to mirror tumor characteristics and response to particle irradiation in recurrent HGG (rHGG).

**Methods:**

^18^F-FET-PET data from n = 43 patients with primary glioblastoma (pGBM) and n = 33 patients with rHGG were assessed. pGBM patients were irradiated with photons and sequential proton/carbon boost, and rHGG patients were treated with carbon re-irradiation (CIR). WBT (Illumina HumanHT-12 Expression BeadChips) lbx was available for n = 9 patients from the rHGG cohort. PET isocontours (40%–70% SUVmax, 10% steps) and MRI-based treatment volumes (MRIvol) were compared using the conformity index (CI) (pGBM, n = 16; rHGG, n = 27). Associations with WBT lbx data were tested on gene expression level and inferred pathways activity scores (PROGENy) and from transcriptome estimated cell fractions (CIBERSORT, xCell).

**Results:**

In pGBM, median SUVmax was higher in PET acquired pre-radiotherapy (4.1, range (R) 1.5–7.8; n = 20) *vs.* during radiotherapy (3.3, R 1.5–5.7, n = 23; p = 0.03) and in non-resected (4.7, R 2.9–7.9; n = 11) *vs.* resected tumors (3.3, R 1.5–7.8, n = 32; p = 0.01). In rHGG, a trend toward higher SUVmax values in grade IV tumors was observed (p = 0.13). Median MRIvol was 32.34 (R 8.75–108.77) cm^3^ in pGBM (n = 16) and 20.77 (R 0.63–128.44) cm^3^ in rHGG patients (n = 27). The highest median CI was observed for 40% (pGBM, 0.31) and 50% (rHGG, 0.43, all tumors) isodose, with 70% (40%) isodose in grade III (IV) rHGG tumors (median CI, 0.38 and 0.49). High SUVmax was linked to shorter survival in pGBM (>3.3, p = 0.001, OR 6.0 [2.1–17.4]) and rHGG (>2.8, p = 0.02, OR 4.1 [1.2–13.9]). SUVmax showed associations with inferred monocyte fractions, hypoxia, and TGFbeta pathway activity and links to immune checkpoint gene expression from WBT lbx.

**Conclusion:**

The benefits of ^18^F-FET-PET imaging on gross tumor volume (GTV) definition for particle radiotherapy warrant further evaluation. SUVmax might assist in prognostic stratification of HGG patients for particle radiotherapy, highlights heterogeneity in rHGG, and is positively associated with unfavorable signatures in peripheral whole-blood transcriptomes.

## Introduction

Despite intensive research and a multimodal treatment approach, the prognosis for glioblastoma (GBM) and recurrent high-grade glioma (rHGG) remains poor (1, 2). Therapy of treatment-naïve GBM consists of maximal safe resection followed by radiotherapy with concomitant and adjuvant temozolomide chemotherapy (3), which may be accompanied by Tumor-Treating Fields (4). For recurrent disease, no standard therapy has been defined; however, resection, radiotherapy (5), and chemotherapy (6) are frequently delivered.

Particle therapy might be a promising treatment strategy for glioma patients. It allows for better normal tissue sparing due to higher physical dose conformity (7) and delivers a higher relative biological effectiveness (RBE) as compared to photon radiotherapy (RBE of 1.87–3.44) (8). Preclinical data suggest benefits from carbon ion radiotherapy (CIR) through improved cell killing of glioma tumor cells in a hypoxic milieu, reduction of angiogenesis, and overcoming a local immunosuppressive milieu (9). In line with these findings, the first clinical data show promising results (10–13).

Metabolic imaging with (18)F-fluoro-ethyl-tyrosine positron emission tomography (18F-FET-PET) has been proposed to help delineate target volumes in glioma (14), which is currently mainly based on contrast-enhanced T1-weighted MRI. It has been hypothesized that 18F-FET-PET is able to detect aggressive tumor subregions and lesions beyond contrast enhancement in MRI (15–17). However, evaluation of 18F-FET-PET-positive isocontour-defined volumes and gross tumor volumes (GTVs) in 26 rHGG patients revealed only low conformity between both volumes with maximum conformities of 0.42–0.51 observed at isocontour 40% (18). A precise algorithm for the integration of 18F-FET-PET into treatment planning is lacking and the subject of ongoing research (19). In addition to target volume definition, 18F-FET-PET imaging has been evaluated for its prognostic value in grade II–IV gliomas (20) and rHGG (18): a poorer outcome for higher tracer uptake has been described in both studies.

Whole-blood transcriptome (WBT) liquid biopsy (lbx) data have been used as a surrogate to monitor disease states (21, 22). Different blood components have the potential to yield information about the tumor with minimal intervention, i.e., circulating tumor cells, or other fractions of blood cells, which might be exposed to a number of transcriptome-altering factors (e.g., irradiation, drug treatments, and transversion through tumor tissue and modulation by cytokines). We recently reported the feasibility of WBT lbx to monitor response to treatment (particle irradiation in rHGG) and to mirror tumor characteristics (23). We therefore aim to evaluate if tracer uptake is linked to a specific whole-blood transcriptome fingerprint.

With this study, we aimed to investigate the value of 18F-FET-PET for tumor delineation in treatment planning and outcome prediction in primary glioblastoma (pGBM) and rHGG treated with particle radiotherapy and to assess a link between WBT lbx readouts and 18F-FET-PET-based tumor metabolic activity quantification.

## Methods

### Ethics approval and consent to participate

All patients consented to participate in this study, and ethical approval was obtained by the IRB-Ethics Committee of the Medical Faculty of Heidelberg University (approval numbers S-421/2015 and S-540/2010).

### Study cohort and radiotherapy

n = 76 patients with pGBM (n = 43) or rHGG (n = 33) were included in this study. All patients underwent high-precision charged particle beam radiotherapy (RT) at the Heidelberg Ion Beam Therapy Center (HIT) between 2010 and 2017. Clinical data were retrospectively recorded in the institute’s own database.

Patients with pGBM were treated in or analogous to the CLEOPATRA trial (24) for incompletely resected primary glioblastoma (residual macroscopic tumor visible at least on MRI). In a two-stage target volume concept, they received a standard postoperative fractionated photon RT up to a cumulative dose of approx. 50 Gy to the contrast-enhanced areas in T1-weighted magnetic resonance tomography (MRT), the T2-hyperintense areas, and the resection cavity, followed by a proton (5 × 2 Gy) or carbon ion (6 × 3 Gray equivalents (GyE)) boost to the macroscopic tumor remnant.

Patients with rHGG (n = 33) were treated in or according to the CINDERELLA trial (10) and received CIR at a dose of 10 to 16 × 3 GyE to the macroscopic tumor. The macroscopic tumor was defined based on contrast enhancement in T1-weighted MRI both for primary and recurrent tumors. As per the study protocol, information from 18F-FET-PET imaging could be optionally considered for the target volume definition of particle RT.

For pGBM, 18F-FET-PET information was not considered for photon RT treatment planning but could be used for particle boost delineation. In the rHGG cohort, 18F-FET-PET information was not considered for GTV delineation but was included in clinical target volume (CTV) delineation; i.e., areas with high tracer uptake were effectively irradiated with CIR. No patient received particle RT in both the primary and recurrent settings.

A static amino acid 18F-FET positron emission tomography (PET) CT scan was available before the start of particle RT for every patient. Tracer uptake and survival analyses were performed on the entire cohort (n = 76). For analyses on the impact of 18F-FET-PET on tumor delineation (see section “Imaging processing and analysis”) in pGBM patients, 18F-FET-PET-derived isocontours were compared to MRI-based GTV for photon RT. Analyses were carried out for n = 16 (37%) pGBM patients. N = 27 patients could not be included due to one or more of the following reasons: photon RT was delivered at another institution, and RT plans were not available for further analyses (n = 14); photon RT planning MRI was not extractable from the institution’s picture archiving and communication system (PACS), i.e., imaging performed at another institution (n = 8); PET showed unspecific tracer uptake without significant evidence of residual tumor (n = 6); PET was carried out >50 days prior to photon RT start (n = 2).

N = 27 (82%) patients with rHGG were included in analyses on the impact of 18F-FET-PET on tumor delineation. 18F-FET-PET-derived isocontours were compared to MRI-based GTV. N = 6 patients could not be included because CIR planning MRI was not extractable from the institution’s PACS (n = 3), because PET was carried out >50 days prior to CIR start (n = 1), which showed uncharacteristic low tracer uptake (n = 1), or imaging was performed at another institution and without a corresponding CT dataset needed for further data procession (n = 1).

Since a relevant proportion of patients could not be included in the analyses of PET vs. MRI-based treatment volumes, it was evaluated whether the characteristics of the enrolled patients were different from those excluded (**Supplementary Tables 3, 4**).

MRI showing tumor progression after particle (re)RT was available for n = 34 (79%) pGBM patients and n = 25 (76%) rHGG patients. Recurrence patterns were analyzed in a semiquantitative approach classifying tumors into one or more of the following categories: 1) recurrence in the area of initial contrast uptake in T1-MRI/MRI-based treatment volume, 2) recurrence in the area of increased PET signal, and 3) distant recurrence.

Whole-blood transcriptome data (Illumina HumanHT-12 Expression BeadChips) were available for n = 9 patients of the rHGG cohort participating in a liquid biopsy study for the effect of CIR in rHGG (for details, see [1]). Blood samples were collected before the start of CIR and at different time points after CIR (see **Supplementary Figure 1**).

### Imaging data

Image data consist of treatment planning MRI and static 18F-FET-PET/CT scans. In pGBM patients, 18F-FET-PET imaging was performed at different time points: before surgery (n = 3), between two surgeries (n = 1, whereby both procedures consisted of a biopsy), between surgery and start of photon RT (n = 16), and during photon RT (n = 23). In rHGG patients, PET images were acquired before CIR in all cases (n = 33), with one patient having had a partial tumor resection between PET acquisition and RT start. Most PET/CT scans were performed with Biograph 6 (n = 61). Other PET/CT devices used were Biograph 64 (n = 5), Biograph mCT Flow (n = 4), Biograph HiRes Model 1080 (n = 2), Biograph TruePoint Model 1094 (n = 2), and Biograph 40 (n = 1; all Siemens Healthineers, Erlangen, Germany) and Gemini TF 16 (n = 1; Philips Medical Systems, Hamburg, Germany).

### Imaging processing and analysis

For analyses of PET imaging data, the standardized uptake value (SUV) was determined. The required parameters (injected activity, injection time, acquisition time, and body weight) were extracted from the static PET DICOM files with Loni Inspector (V 2.11, University of Southern California Mark and Mary Stevens Neuroimaging and Informatics Institute, Los Angeles, USA) software. Median injected activity was 180 (R 124–314) MBq and 2.2 (R 1.39–4.24) MBq/kg body weight for pGBM patients and 198 (R 130–270) MBq and 2.49 (R 1.69–4.15) MBq/kg body weight for rHGG patients. The median time from injection to image acquisition was 12 min (R 5–42) in pGBM patients and 11 min (R 2–41) in rHGG patients.

SUV was determined voxel-wise for all patients using the software MITK (The Medical Imaging Interaction Toolkit, www.mitk.org) (18, 25). In the next step, a large region of interest (ROI) enclosing the primary or recurrent tumor was defined. If required, structures with increased tracer uptake such as vessels and basal ganglia were omitted in ROI delineation. The SUVmax of the ROI was extracted. Next, a maximum standardized uptake ratio (SURmax) was determined for each tumor. SURmax was calculated as the quotient of SUVmax of the ROI and mean SUV of a reference region. The reference region represents the background signal and was drawn in the contralateral hemisphere or, in case of a tumor near the midline, in the unaffected anterior or posterior part of the brain, encompassing both gray and white matter (26).

18F-FET-PET-based tumor volumes were generated as isocontours from SUVmax in increments of 10 from 40% to 70% within the ROI encompassing the tumor. PET images were registered to the treatment planning MRI (rigid registration). To assess differences in PET-derived isocontours (PETvol) and MRI-derived RT treatment volumes (MRIvol), different 3D structures were created and analyzed (see **Supplementary Table 2**). Concordance between volumes was quantified using the conformity index (CI = intersection of PETvol and MRIvol divided by their union). The isocontour with the largest CI was identified as the “best matching isocontour”.

### Whole-blood transcriptome profiling

RNA was extracted from whole blood (PAX gene blood RNA tubes), using the PAXgene Blood RNA Kit (Qiagen, Valencia, CA, USA). Expression was quantified from 200 ng of quality-controlled RNA (Bioanalyzer, Agilent) on Illumina HumanHT-12 Expression BeadChip array in the genomics Core Facility DKFZ (Heidelberg, Germany). xCell (27) and PROGENy (28) were used to estimate cell type fractions and pathway activities from transcriptome data. Kyoto Encyclopedia of Genes and Genomes (KEGG) pathway analysis was conducted with enrichR (29).

### Statistical analysis

Statistical analyses were conducted with IBM SPSS Statistics Version 27 (IBM, Armonk, New York, NY, USA) and R v 4.0.5 (R 30). Time-to-event analyses were conducted with Cox-PH models and parametric survival models assuming log-logistic distribution with the survival (31) and dataAnalysisMisc packages (32). Survival was calculated from the onset of photon RT to progression or death in pGBM and from the onset of CIR to progression or death in rHGG patients. Patients lost to follow-up (FU) were censored at the time point of the last contact. Differences were tested using non-parametric methods (Wilcoxon test, rankFD (33), t-tests, and linear models. Robust p-values were computed using the robustbase package (34). Random forest analyses were conducted with the randomForest package (35). The significance level is set to α = 0.05 (two-sided) if not stated otherwise. The Benjamini–Hochberg procedure was used for multiplicity adjustment (false discovery rate (FDR)) if not stated otherwise.

## Results

### Patient characteristics

**Figure 1:**
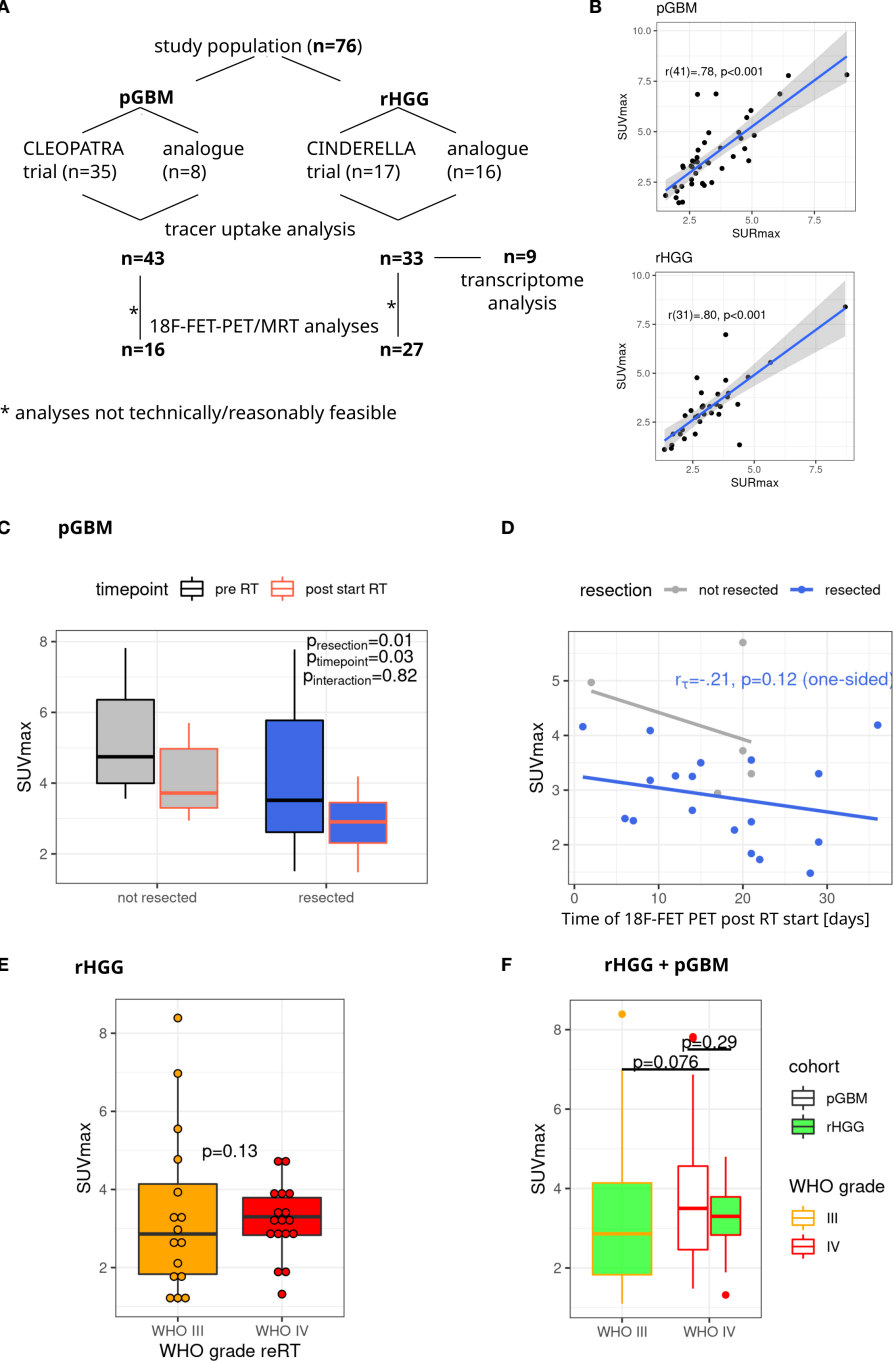
Study cohort and tracer uptake in pGBM and rHGG. **(A)** Overview of the study cohort. **(B)** A strong correlation (r = 0.80) is observed between SUVmax and SURmax values (Pearson’s correlation). **(C)** SUVmax in pGBM depending on resection status and time point after RT start. Non-parametric model analysis (rankFD). **(D)** SUVmax values after irradiation start. Linear model fit, Kendall’s tau for resected samples (one-sided p-value). **(E)** SUVmax in rHGG, one-sided p-value, robust p-value (robustbase). **(F)** SUVmax in grade III and IV tumors. One-sided robust p-value (robustbase). pGBM, primary glioblastoma; rHGG, recurrent high-grade glioma; RT, radiotherapy.

An overview of the cohort, treatment, and patients’ characteristics is shown in **Table 1** and **Figure 1A**.

**Table 1:** Combined table of treatment and patient characteristics for the pGBM (n = 43) and rHGG (n = 33) cohorts.

Feature	Specification	pGBM n (%)	rHGG n (%)
Sex	Male	30 (70)	21 (64)
	Female	13 (30)	12 (36)
Age at initial diagnosis [years]	pGBM: 21–64/rHGG: 16–41	33 (77)	16 (48)
	pGBM: 65–75/rHGG: 42–67	10 (23)	17 (52)
	Median (range)	58 (21–75)	42 (16–67)
Age at CIR [years]	22–64	—–	29 (88)
	65–71	—–	4 (12)
	Median (range)	—–	54 (22–71)
Karnofsky Performance Score* [%]	60–80	10 (23)	7 (21)
	90–100	28 (56)	25 (76)
	N/A	5 (12)	1 (3)
	Median (range)	90 (60–100)	90 (60–100)
Tumor localization	pGBM: unifocal/rHGG: local^+^	38 (88)	30 (91)
	pGBM: multifocal/rHGG: distant^+^	5 (12)	3 (9)
Time from first course of RT to CIR [months]	7–19	—–	16 (48)
	23–208	—–	17 (52)
	Median (range)	—–	23 (7–208)
WHO grade primary tumor	II	0 (0)	10 (30)
	III	0 (0)	8 (24)
	IV	43 (100)	15 (76)
IDH status	Mutation (R132H)	0 (0)	—–
	Wild type	28 (65)	—–
	N/A	15 (35)	—–
MGMT promoter methylation status	Methylated	5 (12)	—–
	Hypomethylated	11 (25)	—–
	N/A	27 (63)	—–
WHO grade recurrence	III	—–	16 (48)
	IV	—–	17 (52)
Maximum extent of surgery	Biopsy	10 (23)	4 (12)
	Resection	33 (77)	29 (88)
	Partial resection	13 (30)	N/A
	Subtotal resection	20 (47)	N/A
Time from last surgery to ^18^F-FET-PET	<5 years	—–	27 (82)
	>5 years	—–	6 (18)
	Median (range) [months]	—–	12 (0–289)
Time from initial RT to ^18^F-FET-PET	<5 years	—–	23 (70)
	>5 years	—–	10 (30)
	Median (range) [months]	—–	22 (6–208)
Tumor progression before RT	Yes	5 (12)	—–
	No	38 (88)	—–
Re-resection performed	Yes	—–	20 (47)
	No	—–	18 (55)
Particle (re)RT	pGBM: protons, 5 × 2 Gy/rHGG: 30–33	26 (60)	14 (42)
	pGBM: carbon ions, 6 × 3 GyE/rHGG: 36–45	17 (40)	19 (58)
	rHGG: median (range)	—–	36 (30–45)
PTV CIR [ml]	5.74–80.82	—–	16 (48)
	83.88–242.44	—–	16 (48)
	N/A	—–	1 (3)
	Median (range)	—–	82.35 (5.74–242.44)
Concurrent chemotherapy	Yes	43 (100)	3 (9)
	No	0 (0)	30 (91)
Follow-up (FU)	FU data available for	37 (86)	31 (94)
	Median FU (range)	7 (1–36)	9 (1–79)

Parameters are presented in absolute numbers and percentages related to the respective cohort.

pGBM, primary glioblastoma; rHGG, recurrent high-grade glioma; CIR, carbon re-irradiation; RT, radiotherapy; PTV, planning target volume; N/A, not available.

*rHGG: CIR.

^+^rHGG: in relation to primary tumor.

The median age at diagnosis of pGBM was 58 years (R 21–75), with a median Karnofsky Performance Score (KPS) of 90% (R 60–100). Prior to RT, 33 (77%) patients underwent resection (partial resection, 13 (30%); subtotal resection, 20 (47%)), and 10 patients (23%) underwent biopsy only. The sequential particle boost was delivered with protons (5 × 2 Gy) in 26 cases (60%) and CIR (6 × 3 GyE) in 17 cases (40%). Tumor tissue was available from all pGBM patients, and the diagnosis of glioblastoma was based on the 2007 WHO Classification of Tumors of the Central Nervous System (36) in most cases (n = 41, 95%). Two patients were classified according to the 2016 WHO classification (37). IDH1 status was available in 28 cases (65%), all of which showed IDH1-wild-type glioblastoma. Median overall survival (OS) was 16.2 months (95% CI 13.3–19.7). Median progression-free survival (PFS) was 6.4 months (95% CI 5.2–7.8).

In the rHGG cohort, the median age at the time of CIR was 54 (R 22–71) years, and the median KPS was 90% (R 60–100). For rHGG patients, tumor tissue was available for n = 15 (45%) patients at the time point of recurrence. Tumor classification was mostly based on the 2007 WHO Classification (n = 14, 93%) with one case being classified according to the 2016 WHO Classification. In n = 18 (55%) patients, where no biopsy or resection was performed at recurrence, the diagnosis was based on imaging criteria suggestive of rHGG (contrast medium enhancement) and an interdisciplinary consensus (tumor board). At disease recurrence, 16 (48%) patients had a WHO grade III, and 17 (52%) patients had a WHO grade IV tumor. The initial diagnosis was determined histologically in all rHGG patients. The underlying primary tumor was low-grade glioma (LGG) in n = 10 (30%) and HGG in n = 23 (70%) cases, with n = 8 (24%) grade III vs. n = 15 (45%) grade IV gliomas. In the primary setting, classification was according to the 2007 WHO classification in n = 23 (70%) and according to previous versions in the remaining n = 10 (30%) cases. The median time from the first course of photon RT to CIR was 22 months (R 6–208). Initially, n = 29 (88%) patients underwent tumor resection vs. n = 4 (12%) biopsy only. Re-resection had been performed in 15 cases (45%). The median time between the last resection and 18F-FET-PET was 14 months (R 1–289). CIR was delivered at a median dose of 39 GyE (R 30–45) in 3-GyE fractions. Median OS in rHGG was 17.5 months (95% CI 11.3–27.2) with the date of death known for 23 (70%) patients. PFS was 5.0 months (95% CI 3.4–7.6).

### Tracer uptake in primary glioblastoma and recurrent high-grade glioma

Tracer uptake, as quantified by SUVmax and SURmax, is shown for both cohorts in **Figure 1**. Both parameters show high concordance (**Figure 1A**), and no clear improvement using SURmax in performed analyses was observed (data not shown). Thus, SUVmax-based analyses were retained going forward. Median SUVmax was 3.5 (R 1.48–7.82) in pGBM. 18F-FET-PET tracer uptake was significantly lower in patients with resected tumors and in images acquired after the start of photon RT (**Figure 1B**). There was no interaction between resection status and time point of 18F-FET-PET imaging (p = 0.82, **Figure 1B**). For resected tumors under RT, there was a non-significant association for decreasing 18F-FET-PET uptake if imaging was performed at later stages of RT (**Figure 1C**, p = 0.12, one-sided).

In rHGG, PET images yielded a median SUVmax of 3.09 (R 1.1–8.39). A trend toward higher SUVmax values in grade IV tumors was observed (**Figure 1D**). This trend was preserved after pooling pGBM and rHGG cohorts, with higher SUVmax values in grade IV vs. grade II tumors (p = 0.08) (**Figure 1E**).

### Prognostic value of tracer uptake

**Figure 2:**
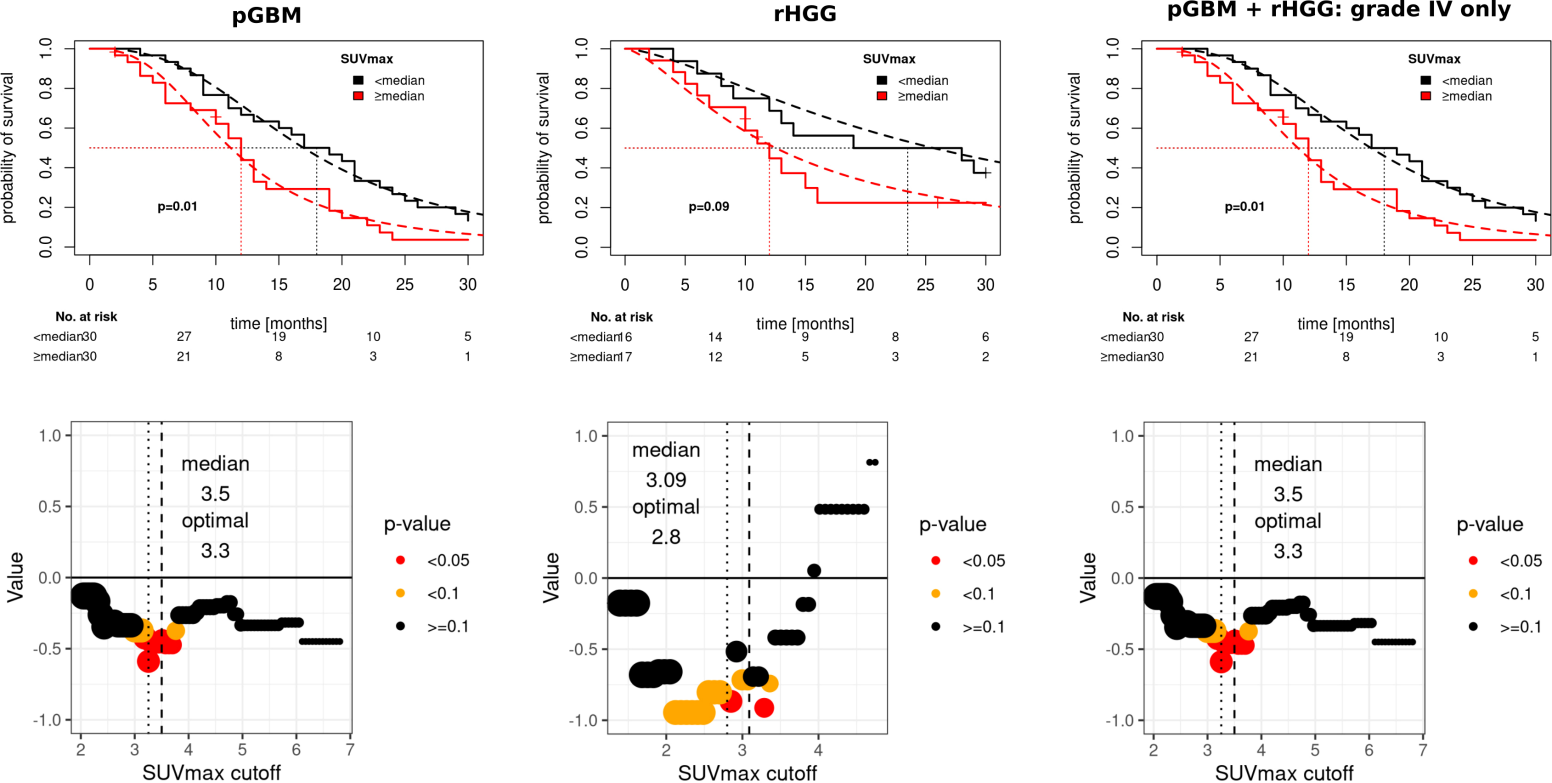
Prognostic value of SUVmax in pGBM and rHGG. Top row: Kaplan–Meier survival curves, log-logistic parametric survival regression fits (dashed line). Wald type p-value. Bottom row: model coefficients for varying cutoffs (bottom row), median (dashed line), and optimal cutoffs (minimal p-value, dotted line). pGBM, primary glioblastoma; rHGG, recurrent high-grade glioma.

To test the prognostic value of SUVmax for OS, patients from both cohorts were split into “higher than median” vs. “lower than median” SUVmax uptake using the median SUVmax value of each cohort as a cutoff (pGBM cutoff, 3.5; rHGG cutoff, 3.09; **Figure 2**, top row). For patients with pGBM, SUVmax ≥ 3.5 was prognostic for OS (p = 0.01), whereas in rHGG, an uptake > 3.09 was associated with a non-significant trend for worsened survival outcomes. A combination of grade IV tumors from pGBM and rHGG confirmed a prognostic value of SUVmax for median cutoff (3.37, **Figure 2**, right). In the pooled grade IV tumors from pGBM and rHGG, median SUVmax (>3.37) was prognostic for worsened OS (p = 0.01). Additionally, we attempted to identify the best prognostic SUVmax threshold by evaluating the impact of varying cutoffs on OS. SUVmax optimal was defined as the cutoff associated with the smallest p-value for OS (**Figure 2**, bottom row). In rHGG, an optimal cutoff of 2.8 was prognostic of outcome (p < 0.05). For pGBM, SUVmax optimal was 3.3. For ranges of cutoffs leading to the separation of groups with p < 0.05 or p < 0.1, see **Supplementary Table 2**.

**Figure 3:**
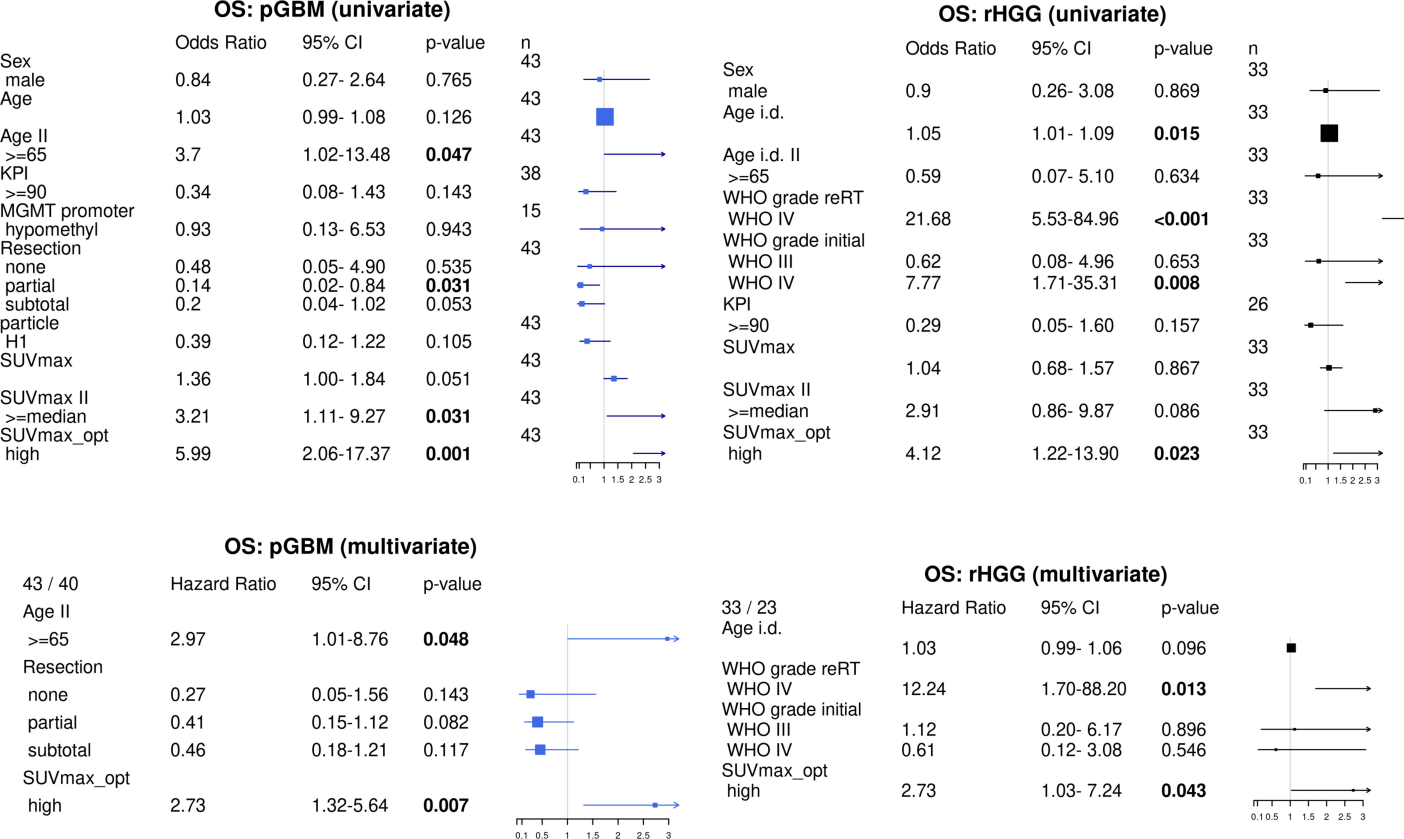
Uni- and multivariate survival analyses in pGBM and rHGG. Univariate analysis: parametric survival model (log-logistic distribution). pGBM: reference level for resection: biopsy. Age i.d.: age at initial diagnosis. Multivariate analysis includes variables significant in univariate analysis, Cox PH survival model. SUVmax_opt: optimal separation (minimal p-value). pGBM, primary glioblastoma; rHGG, recurrent high-grade glioma.

SUVmax as a continuous covariate was significant for worsened OS in pGBM (OR = 1.36, p = 0.051). SUVmax median cutoff and SUVmax optimal cutoff were also prognostic factors for OS (OR = 3.21, p = 0.31 and OR = 5.99, p = 0.001 respectively). Other prognostic variables on the univariate analysis included age (≥ 65 vs. <65 years, OR = 3.7, p = 0.047) and degree of resection, whereby partial resection (OR = 0.14, p = 0.03) and subtotal resection (OR = 0.2, p = 0.053) were associated with improved OS. Multivariate analysis confirmed SUVmax optimal (OR = 2.73, p = 0.007) and age ≥ 65 years (OR = 2.97, p = 0.048) as independent adverse prognostic markers for OS in pGBM (**Figure 3**).

In rHGG, SUVmax as a continuous variable was not prognostic of OS on univariate analysis (**Figure 3**). SUVmax median showed a trend for worsened OS (OR = 2.91, p = 0.086), and SUVmax optimal was prognostic for OS (OR = 4.12, p = 0.023). Additionally, age at initial diagnosis (OR = 1.05, p = 0.015), initial WHO grade IV (HR = 7.8, p = 0.008), and WHO grade IV at CIR (HR = 21.7, p = 0.001) were inversely associated with OS. Multivariate analysis confirmed SUVmax optimal (OR = 2.73, p = 0.042) and WHO grade IV at CIR (OR = 12.24, p = 0.013) as independent prognostic markers for OS in rHGG (**Figure 3**).

SUVmax optimal cutoff was also prognostic for PFS in pGBM (cutoff 2.3, OR 11.9 [95% CI 1.95–71.9], p = 0.007) and rHGG (cutoff, 4.4, OR 4.36 [95% CI 1.15–16.52], p = 0.03). The data are shown in **Supplementary Figure 2**.

### Isocontour concordance with irradiated volume

Comparison of 18F-FET-PET-defined isocontours with MRI-based treatment volumes was carried out for a subset of patients due to the limited availability of data (see **Figure 1A** and “Methods”). Treatment and patients’ characteristics of the subcohorts are shown in **Supplementary Table 3** and SUVmax metrics in **Supplementary Table 4**.

**Figure 4:**
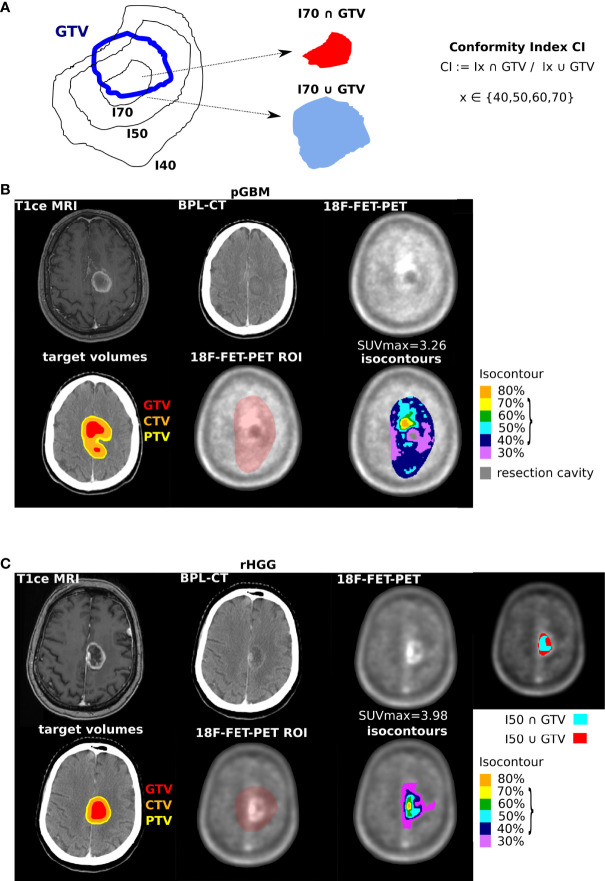
Concordance between ^18^F-FET-PET defined volumes and target volumes. **(A)** Schematics of evaluated volumes with isocontour I (I40%, 50%, and 70% shown as an example) and conformity index (CI) definition. **(B)** Representative images with radiotherapy volumes and isocontours for a patient with primary glioblastoma, from left to right in the first row: MRI at initial diagnosis, planning CT, ^18^F-FET-PET imaging before radiotherapy; in the second row: planning CT with MRI-based tumor volumes (GTV in red, CTV in orange, and PTV in yellow), defined region of interest (ROI) in ^18^F-FET-PET imaging, and ^18^F-FET-PET-based isocontours (30% in purple, 40% in blue, 50% in turquoise, 60% in green, 70% in yellow, and 80% in orange). **(C)** Representative images with radiotherapy volumes and isocontours for a patient with recurrent high-grade glioma, imaging, and color coding similar to panel **(B)** Additional illustration of a representative image for I50 and GTV with intersection of volumes shown in turquoise and union of volumes shown in red. Parentheses, values further evaluated; see **Figure 5**. GTV, gross tumor volume; CTV, clinical target volume; PTV, planning target volume.

**Figure 5:**
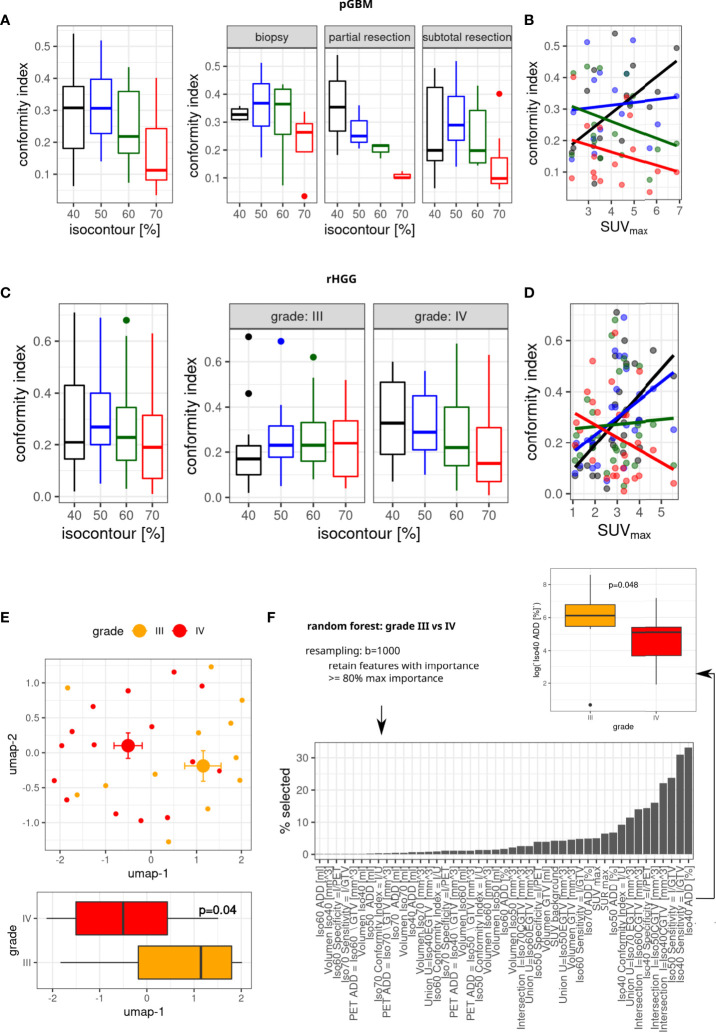
Concordance between GTV and ^18^F-FET-PET isocontours. Conformity index (CI) for different isocontours **(A, C)**, split by degree of resection (pGBM, A, right) and radiographic reRT tumor grade (C, right). **(B, D)** Association between SUVmax and conformity index (linear model fits). **(E)** Umap representation of all n = 45 volumetric features, median, and SE (top part). Bottom part: differences in umap-1 values between tumor grades (linear model Wald type p-value). **(F)** Identification of features separating grade using random forests analysis (top part); distribution of feature frequencies in bottom part, and differences in top-ranked feature is shown on the right (linear model p-value). n = 27 patients. GTV, gross tumor volume; pGBM, primary glioblastoma.

GTV was utilized as MRIvol for pGBM in all 16 cases. In rHGG, GTV was used in n = 24 (89%) and CTV in 3 (11%) cases, where GTV was not defined but where areas of high tracer uptake morphologically corresponded to contrast enhancement in T1-MRI. Concordance between 18F-FET-PET isocontours and MRIvol was assessed with CI (**Figures 4**, **5**) and Dice coefficient (**Supplementary Figure 3**). **Figure 4A** illustrates the calculation of CI for a given GTV volume and a range of isocontours (Ix). **Figure 4B** shows images of a patient from the pGBM cohort and **Figure 4C** of a patient from the rHGG cohort with resection of the initial and no resection of the recurrent tumor.

The highest conformity was observed for isocontour 40% in pGBM and isocontour 50% in rHGG (**Figures 5A, C**). Stratification by resection status in pGBM showed for biopsy and subtotal resection high conformity for 50% isocontour and only for partial resection isocontour 40% showed the highest median CI. In rHGG, conformity decreased for higher isocontours in grade IV tumors. The opposite was observed in grade III rHGG whereby higher conformity was seen for I70 (**Figures 5A, C**). Finally, the relationship between CI and SUVmax was investigated in each group. In pGBM (**Figure 5B**), the CI was negatively associated with SUVmax at higher isodoses (I70: red line). In contrast, CI was positively correlated with increasing SUVmax for isodoses 50 (blue line) and 40 (black line). Similar correlations were seen in rHGG (**Figure 5D**).

In pGBM/rHGG, the median volume of “best matching isocontour” was 22.59 (R 5.72–192.68)/24.35 (R 2.42–1,407.04) cm3. Its union with GTV resulted in a median 26% (R 3–328)/37% (R 0.2–4836) increase in volume, with the median absolute volume added by “best matching isocontour” being 8.35 (1.37–159.04)/6.3 (R 0.13–73.03) cm3. Absolute volumes for each patient are visualized in **Supplementary Figure 4**

### Radiographic features are associated with recurrent high-grade glioma grade

Next, volumetric parameters in rHGG were evaluated (intersections of isocontour volumes and MRI-based target volumes) as well as tracer uptake metrics (SUVmax, SURmax; see the full list in **Supplementary Table 2**). Radiographic features separated patients with rHGG into two separate populations on the two-dimensional umap representation (**Figure 5E**). This separation corresponded to rHGG tumor grade (**Figure 5E**, top part), with umap-1 showing significantly different values between grades (p = 0.04, **Figure 5E**, bottom part). Next, random forest analyses with resampling were used to identify which features dominate separation by grade (**Figure 5F**, bottom part). Random forest selected features in increasing order of importance. Interestingly, the top-ranked features were those features associated with isocontour 40/50. In contrast, SURmax and SUVmax were only ranked in positions 11 and 12. Iso 40 ADD was the top-ranked feature and was enough to separate patients according to grade (**Figure 5F**, right).

### Patterns of recurrence

Assessment of recurrence patterns for the pGBM cohort (subcohort of n = 34 patients with follow-up imaging showing progress), where 18F-FET-PET was only utilized for boost delineation, showed n = 21 (62%) local recurrences, n = 5 (15%) distant recurrences, and simultaneous local and distant recurrences for n = 8 (24%) patients at first progression. In four cases, recurrences occurred in areas with increased tracer uptake (SUVmax: 3.25, 3.51, 5.7, and 7.82), one of which did not show contrast enhancement on MRI and the remaining three being partially included in photon-RT MRIvol.

In rHGG (subcohort of n = 25 patients with follow-up imaging showing progress), where 18F-FET-PET was used for CTV delineation, no local recurrence was observed in 18F-FET-PET-positive/MRI T1ce-negative areas. N = 3 (12%) patients showed distant recurrences, n = 17 (68%) local recurrences, and n = 5 (20%) simultaneous local and distant recurrences at first progression.

### SUVmax correlates with whole-blood transcriptomes

**Figure 6:**
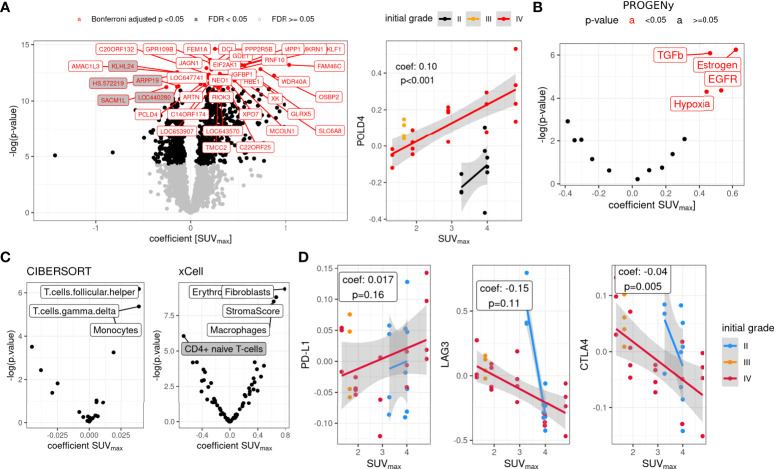
Whole-blood transcriptome associations with SUVmax in the rHGG cohort. **(A)** Genes associated with SUVmax, adjusted for initial WHO grade (most variable 10% of genes: median absolute deviation, gray background, coefficient < 0) and representative data for POLD4 (right) with linear fits. **(B)** PROGENy estimated pathway activity from transcriptome data (non-adjusted p-value). **(C)** CIBERSORT and xCell estimated cell fractions associated with SUVmax (adjusted for initial tumor grade, z-scaled data, non-adjusted p-value). **(D)** Association of SUVmax with immune checkpoint gene expression (linear mixed models). n = 9 patients. rHGG, recurrent high-grade glioma.

We assessed if SUVmax correlates with whole-blood transcriptomes. Analysis of the most variable 10% of genes adjusted for initial tumor grade identified 38 genes as being associated with SUVmax (Bonferroni-adjusted p-value <0.05) and 936 genes with an FDR < 0.05 (**Figure 6A**). As an example, representative expression values for POLD4 are shown in **Figure 6A** (top middle), demonstrating an association between gene expression and SUVmax as a function of the initial WHO grade. KEGG pathway enrichment identified Glioma, PI3K pathway components, and specific HLA genes associated with SUVmax (**Supplementary Figure 5**).

Higher SUVmax showed positive associations with hypoxia, TGFbeta, and EGFR transcriptome signatures as inferred by PROGENy (**Figure 6B**). Fractions of cell types, inferred with CIBERSORT and xCell, showed higher fractions of macrophages/monocytes in higher SUVmax tumors and depletion of CD4+ naïve T cells (**Figure 6C**). Finally, the evaluation of immune-checkpoint gene expression showed a trend toward a positive association of SUVmax and PD-L1 and negative associations for LAG3 (p = 0.11) and CTLA4 (p = 0.005) (**Figure 6D**).

## Discussion

In this study, we report on the value of 18F-FET-PET imaging for target volume definition in pGBM treated with photon irradiation with particle boost and rHGG treated with carbon particle therapy, as well as its use for prognosis estimation and a link to minimally invasive whole-blood transcriptome liquid biopsy readouts.

Particle therapy provides an attractive therapeutic option for pGBM (11, 38) and rHGG (11, 38). However, tumors inevitably recur, preferentially in-field in patients treated with conventional photon radiotherapy (39) and at field margins in rHGG patients treated with carbon ions (18). Thus, identification of tumor (sub-)regions that might require intensified treatment as they are more prone to recurrence is crucial. In this study, however, we did not observe a clear preference for local vs. distant recurrences, which might be caused by a much less fine-granular qualitative classification in local vs. distant recurrence.

18F-FET-PET has been proposed to help delineate aggressive tumor regions and lesions not detected on T1 contrast-enhanced (ce) MRI (15–17, 40, 41). In our analyses, we see only a low degree of concordance between 18F-FET-PET and MRI-based target volumes, with the highest observed CIs for isocontours of 40% to 50%. This supports the hypothesis that T1ce MRI and 18F-FET-PET imaging show different aspects of the tumor and that additional information can be obtained by 18F-FET-PET imaging. “Best matching isocontours” were not necessarily larger than MRI-derived treatment volumes; however, we showed that their addition to GTV could likely result in a substantial increase in the treatment volume. A low threshold of I50 also implies a relatively large volume based on 18F-FET-PET, which might lead to intolerable toxicities. In pGBM, this relatively low overlap can be to some extent explained by the inclusion of the resection cavity into the MRI-based target volume; however, in rHGG, the resection cavity was generally not included in the target volumes. In line with these findings, Debus et al. also reported a low CI of 0.42–0.51 at I40% (18). More detailed analyses revealed a positive association of CI with SUVmax up to isocontour 50%; however, resection status and tumor grade had an influence on the optimal isocontour (maximal CI) in our cohorts and should be considered as covariates in future studies.

To further examine the hypothesis that 18F-FET-PET delineates highly aggressive tumor regions, we simultaneously assessed a number of tracer uptake metrics and volumetric features derived from isocontour/target volume intersections in rHGG tumors. We observed a separation of tumors by grade, and random forest analysis identified I40 features as being able to discriminate between tumor grades. The added volume based on I40 is higher in grade III tumors vs. grade IV tumors, and accordingly, the median CI for I40 is lower for grade III vs. IV. Thus, the overlap of 18F-FET-PET and target volumes is higher in grade IV rHGG, thus supporting the hypothesis that 18F-FET-PET may be better suited for more aggressive tumors.

In this line, we found significantly increased tracer uptake for non-irradiated (18F-FET-PET acquisition before RT) as well as non-resected GBM and a trend toward higher SUVmax in GBM. It seems plausible that higher metabolic activity resulting in a higher tracer uptake is observed in untreated and more aggressive tumors.

Tracer uptake as a prognostic factor has been described previously. Gempt et al. reported that a tumor-to-normal ratio (SUVmean tumor/SUVmean background) of 1.88 was best in discriminating OS in patients with primary WHO grade II to IV glioma (40), whereas Sweeney et al. identified SUVmax of 2.6 as the cutoff for survival prognosis in their cohort of primary WHO grade II to IV patients (20). For recurrent WHO grade IV gliomas, Debus et al. reported a poorer prognosis with SUVmax > 2.92 (18).

We confirmed that tracer uptake is associated with prognosis both in the primary (pGBM) and in the recurrent setting (rHGG). Our identified thresholds/threshold ranges leading to prognostic separation are in line with the reported data. Thus, tracer uptake may allow for prognostic stratification independent of the therapeutic situation (primary vs. recurrent tumor). However, determination of a fixed tracer uptake cutoff for prognostication is difficult, as tracer uptake not only is dependent on the tumor itself but also reflects the imaging protocol used. It is also biased by previous therapies, as shown in our analyses, and the prognosis is obviously dependent on antitumor treatment and patient-related factors as well.

In our study, analyses of tracer uptake were based on SUVmax. We also calculated SURmax values, which are the SUVmax values corrected for the appropriate SUV background signal (26), and we observed a high concordance between both metrics. Thus, more complex background-adjusted metrics are not necessarily required.

Finally, using WBT profiling of a subset of the rHGG cohort, we found gene expression differences associated with SUVmax, adjusted for initial tumor grade (stable initial grade WBT signature as recently reported (23)). Among them were genes involved in DNA repair (ATM and POLD4). Pathway activity analysis showed an association of unfavorable pathways with higher SUVmax values (TGFbeta and hypoxia) (9, 42), and inferred monocyte/macrophage fractions were enriched in high SUVmax tumors. In addition, immune checkpoint gene expression showed differential regulation, especially CTLA4 (low expression in high SUVmax tumors). This supports the hypothesis of glioma as a systemic disease, with a pronounced exchange between the tumor and its environment (39).

The main limitation of the present study is the limited number of patients, especially for the whole-blood transcriptome analyses. Here, further independent studies including larger cohorts should be performed to validate our findings. Technical differences (different scanners), missing data, and (molecular) heterogeneity within the group of recurrent high-grade glioma might affect the present results. In addition, dynamic 18F-FET PET might reveal additional insights into tumor metabolism.

## Conclusion

18F-FET PET has a prognostic value in both treatment-naïve glioblastoma and recurrent high-grade glioma. Its value on target volume definition remains less clear, with overall low concordance between 18F-FET-PET and target volumes. High metabolic activity, as quantified by SUVmax, is linked to worse prognosis and unfavorable whole-blood transcriptome liquid biopsy readouts. Our results warrant confirmation in larger, prospective studies.

## Data availability statement

The raw data supporting the conclusions of this article will be made available by the authors, without undue reservation.

## Ethics statement

This study was reviewed and approved by IRB-Ethics Committee of the Medical Faculty of Heidelberg University (approval number S-421/2015 and S-540/2010). The patients/participants provided their written informed consent to participate in this study.

## Author contributions

Conceptualization and methodology: MW, MR, JF, PS, JD, UH, MK, LK, CD, AG, AK, MD, UW, CH-M, SC, AA. Formal analysis: MW, MK. Investigation: MW, MK, CS, UW. Writing - original draft: MW, BT, AG, MK, AA. All authors contributed to the article and approved the submitted version.

## Funding

This research was funded by the German Research Foundation Collaborative Research Center (DFG, SFB 1389, Unite, Project-ID 404521405), EU Predict, and intramural funds of the National Center for Tumor Diseases (NCT).

## Conflict of interest

The authors declare that the research was conducted in the absence of any commercial or financial relationships that could be construed as a potential conflict of interest.

## Publisher’s note

All claims expressed in this article are solely those of the authors and do not necessarily represent those of their affiliated organizations, or those of the publisher, the editors and the reviewers. Any product that may be evaluated in this article, or claim that may be made by its manufacturer, is not guaranteed or endorsed by the publisher.
