# Supplementary figures and images for: The impact of tumor metabolic activity assessed by 18F-FET amino acid PET imaging in particle radiotherapy of high-grade glioma patients
Source: Front Oncol. 2022 Sep 20;12:901390. doi: 10.3389/fonc.2022.901390 (PMC9531169; doi:10.3389/fonc.2022.901390)

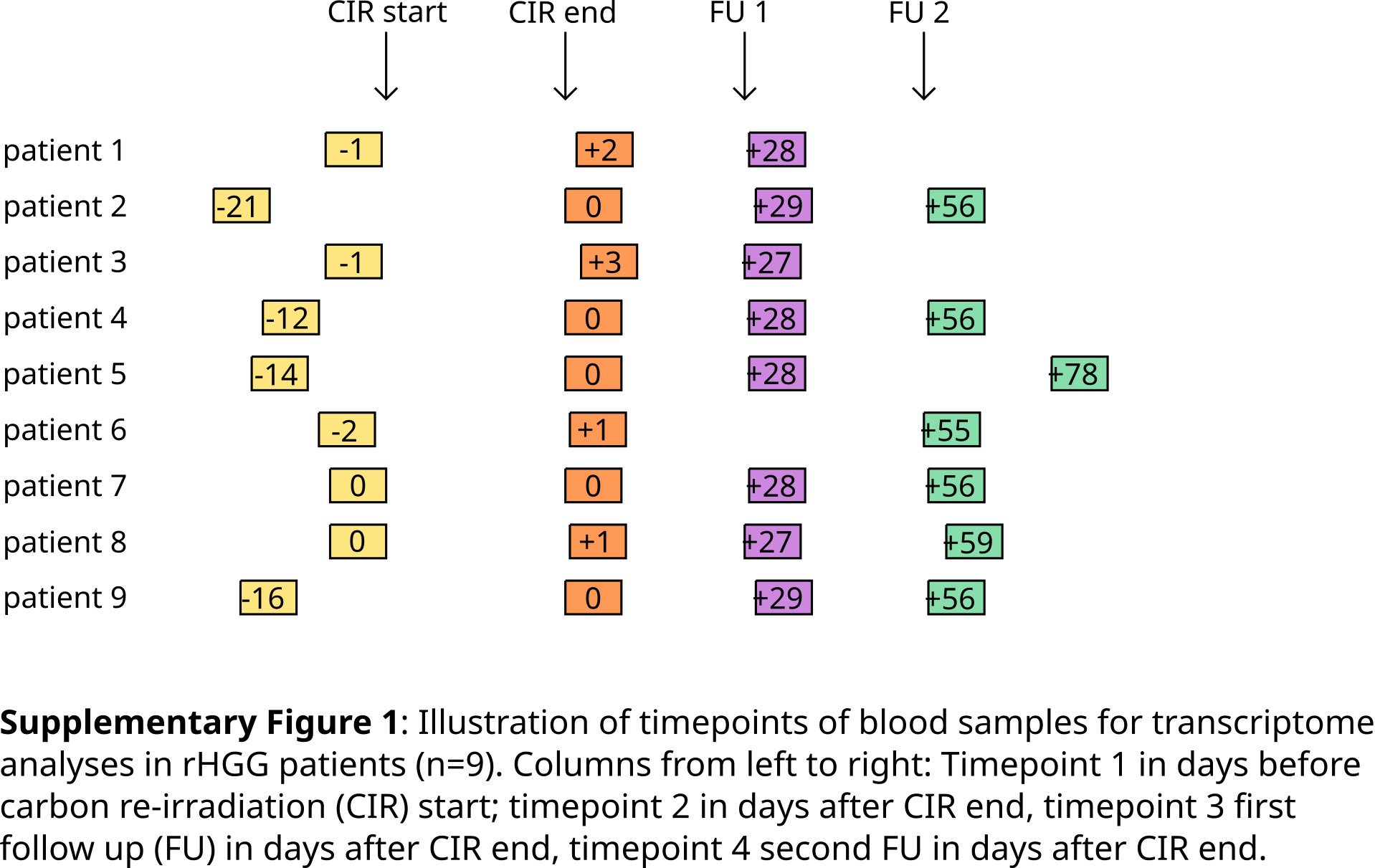

Supplement: Supplementary file 1 [file Image_1.jpeg]

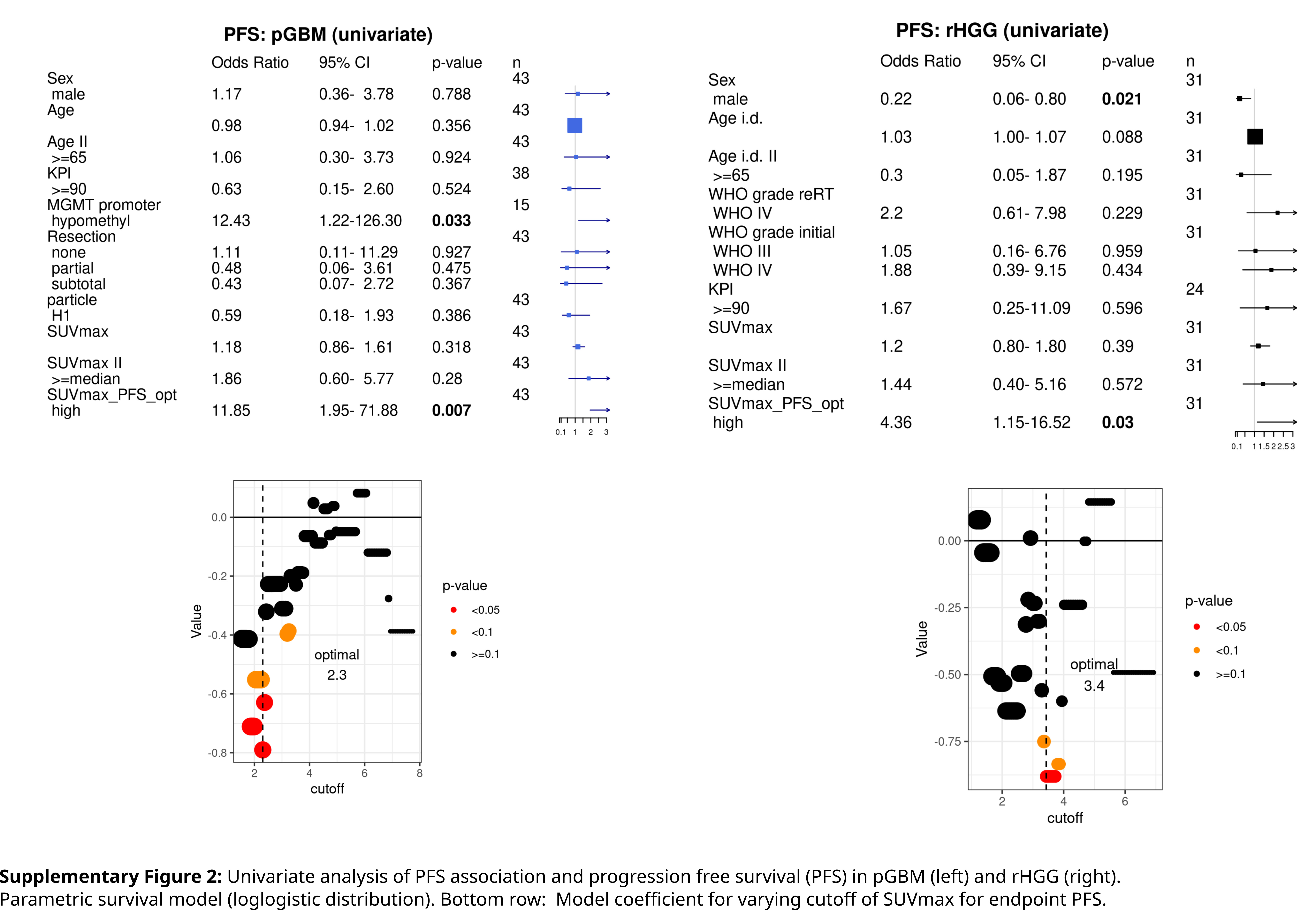

Supplement: Supplementary file 2 [file Image_2.jpeg]

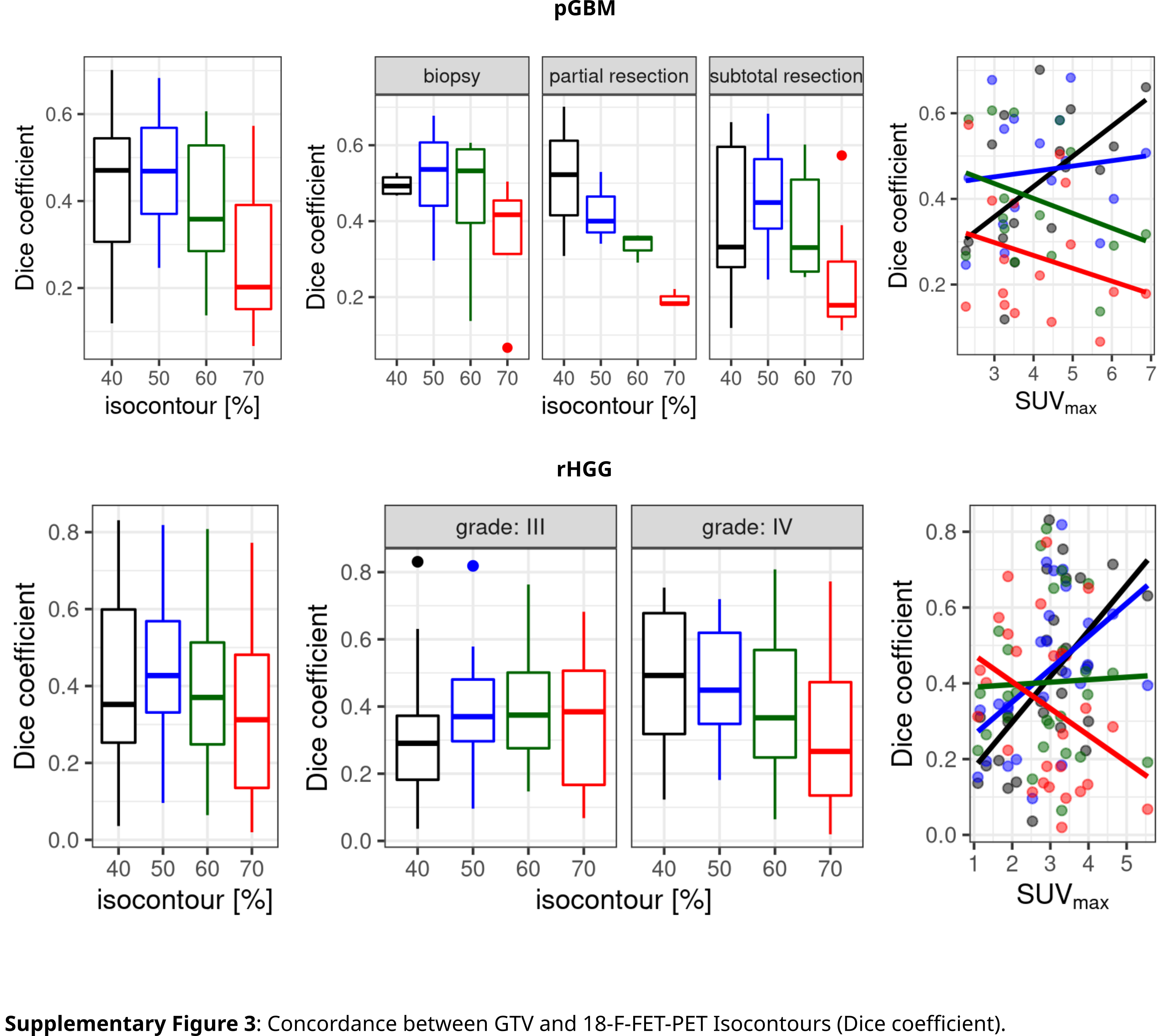

Supplement: Supplementary file 3 [file Image_3.jpeg]

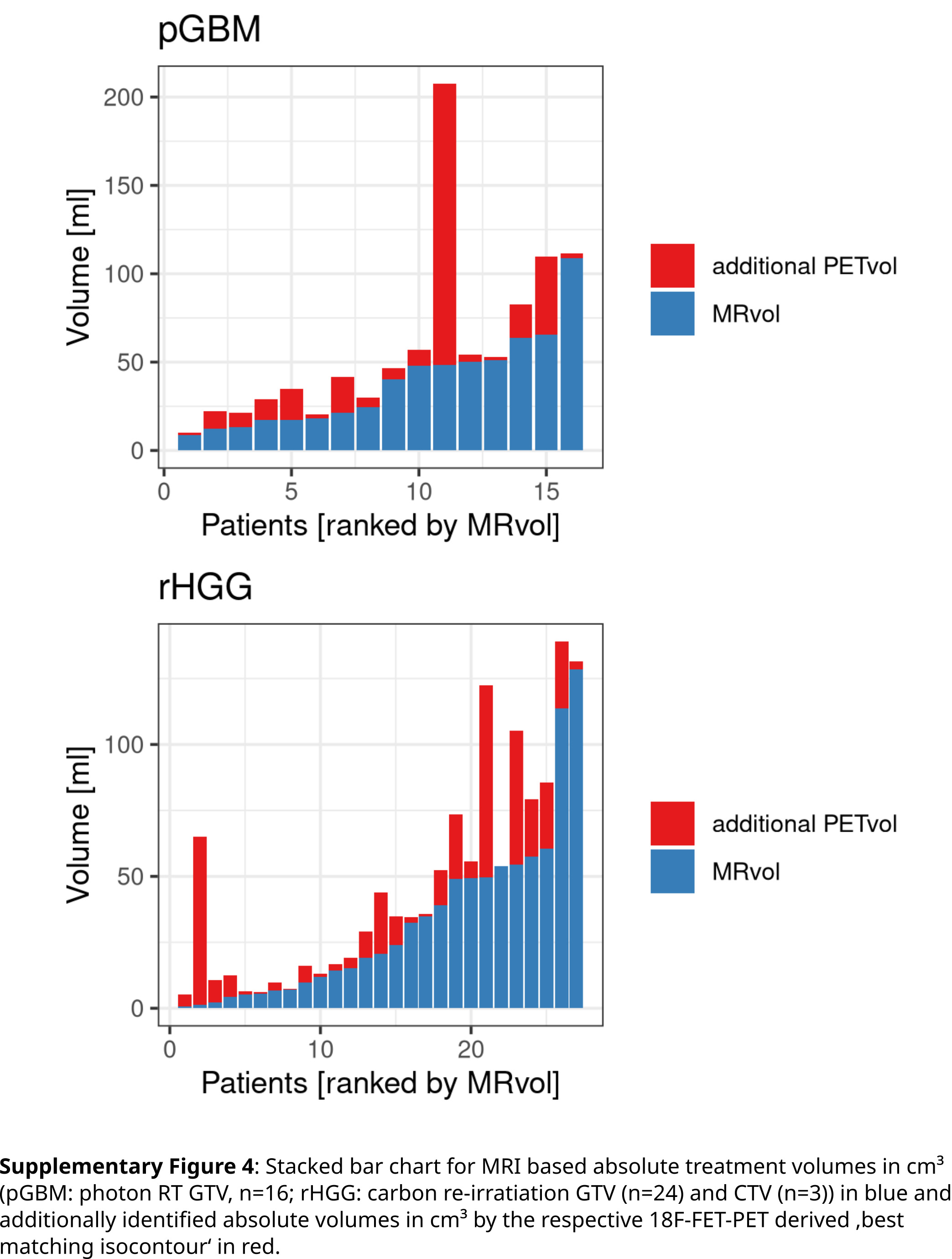

Supplement: Supplementary file 4 [file Image_4.jpeg]

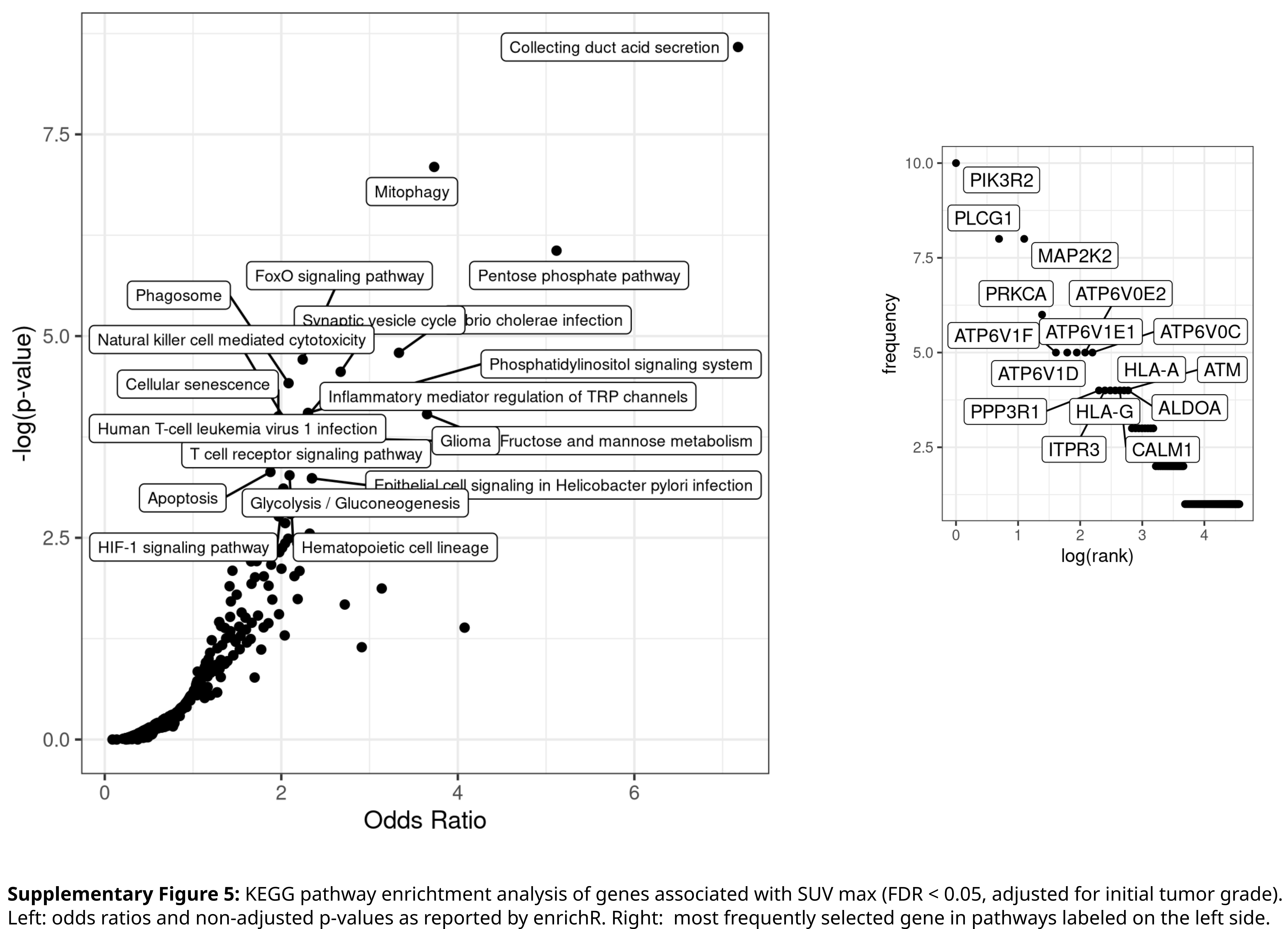

Supplement: Supplementary file 5 [file Image_5.jpeg]
