# Supplementary material for: The impact of tumor metabolic activity assessed by 18F-FET amino acid PET imaging in particle radiotherapy of high-grade glioma patients
Source: Front Oncol. 2022 Sep 20;12:901390. doi: 10.3389/fonc.2022.901390 (PMC9531169; doi:10.3389/fonc.2022.901390)
Supplement: Supplementary file 6 [file Table_1.docx]

# Supplementary Tables

**Supplementary Table 1:** SUVmax cutoffs leading to prognostic separation of the respective subcohorts for p<0.1, parametric survival model, loglogistic distribution. Maximum ranges are reported.

| Cohort | Interval | p-value |
| --- | --- | --- |
| rHGG | | |
|  | 2.12-3.36 | <0.1 |
|  | 2.78-3.29 | <0.05 |
| pGBM |  |  |
|  | 3.26-6.87 | <0.1 |
|  | 3.26-4.08 | <0.05 |

**Supplementary Table 2:** Definition of 3D-structures for assessment of PET and MRI-derived tumor volumes with PETvol being the respective created isocontour from SUVmax in increments of ten from 30% to 80% and MRIvol being photon RT GTV for pGBM and carbon ion reRT GTV in 24 cases or CTV in three cases where GTV was not available for rHGG.

| Structure | Definition |
| --- | --- |
| Intersection | PETvol ∩ MRIvol |
| Union | PETvol ∪ MRIvol |
| Conformity Index (“CI”) | intersection/union |
| Treatment volume coverage (“sensitivity”) | intersection/MRIvol |
| PET volume addition (“PET ADD”) | 1-(intersection/PETvol) |

**Supplementary Table 3:** Combined table of treatment and patient characteristics for pGBM (n=16) and rHGG (n=27) patients included in PET/treatment volume analyses. Parameters are presented in absolute numbers and percentages related to the respective cohort.

| Feature | Specification | pGBM n(%) | rHGG n (%) |
| --- | --- | --- | --- |
| Sex | male | 11 (69) | 18 (67) |
|  | female | 5 (31) | 9 (33) |
| Age at initial diagnosis [years] | pGBM:29-64/rHGG:16-41 | 14 (88) | 13 (48) |
|  | pGBM:65-70 /rHGG: 42-67 | 2 (13) | 14 (52) |
|  | Median (Range) | 50 (29-70) | 42 (16-67) |
| Age at CIR [years] | 22-64 | ----- | 24 (89) |
|  | 65-71 | ----- | 3 (11) |
|  | Median (Range) | ----- | 54 (22-71) |
| Karnofsky Performance Score* [%] | 60-80 | 2 (13) | 6 (22) |
|  | 90-100 | 12 (75) | 20 (74) |
|  | N/A | 2 (13) | 1 (4) |
|  | Median (Range) | 90 (70-100) | 90 (60-100) |
| Tumor localization | pGBM:unifocal/rHGG:local^+^ | 14 (88) | 24 (89) |
|  | pGBM:multifocal/rHGG:distant^+^ | 2 (13) | 3 (11) |
| Time from first course of RT to CIR | 7-19 | ----- | 13 (48) |
| [months] | 23-162 | ----- | 14 (52) |
|  | Median (Range) | ----- | 23 (7-162) |
| WHO grad primary tumor | II | 0 (0) | 8 (30) |
|  | III | 0 (0) | 6 (22) |
|  | IV | 16 (100) | 13 (48) |
| IDH status | mutation (R132H) | 0 (0) | ----- |
|  | wild type | 13 (81) | ----- |
|  | N/A | 3 (19) | ----- |
| MGMT promoter methylation status | methylated | 2 (13) | ----- |
|  | hypomethylated | 1 (6) | ----- |
|  | N/A | 13 (81) | ----- |
| WHO grade recurrence | III | ----- | 12 (44) |
|  | IV | ----- | 15 (56) |
| Maximum extent of surgery | biopsy | 4 (25) | 4 (15) |
|  | resection | 12 (75) | 23 (85) |
|  | partial resection | 3 (19) | N/A |
|  | subtotal resection | 9 (56) | N/A |
| Time from last surgery to 18F-FET-PET | <5 years | ----- | 23 (85) |
|  | >5 years | ----- | 4 (15) |
|  | Median (Range) [months] | ----- | 12 (0-260) |
| Time from initial RT to 18F-FET-PET | <5 years | ----- | 19 (70) |
|  | >5 years | ----- | 8 (30) |
|  | Median (Range) [months] | ----- | 22 (6-162) |
| Tumor progression before RT | yes | 2 (13) | ----- |
|  | no | 14 (88) | ----- |
| Re-resection performed | yes | ----- | 11 (41) |
|  | no | ----- | 16 (59) |
| Particle RT/CIR | pGBM: protons, 5 x 2 Gy/rHGG:30-33 | 10 (63) | 13 (48) |
|  | pGBM: carbon ions, 6 x 3 GyE/rHGG:36-45 | 6 (38) | 14 (52) |
|  | rHGG: Median (Range) | ----- | 36 (30-45) |
| PTV CIR [ml] | 5.74-80.82 | ----- | 14 (52) |
|  | 85.75-242.44 | ----- | 12 (44) |
|  | N/A | ----- | 1 (4) |
|  | Median (Range) | ----- | 77.32 (5.74-242.44) |
| Concurrent chemotherapy | yes | 19 (100) | 1 (4) |
|  | no | 0 (0) | 26 (96) |
| Follow-up (FU) | FU data avaliable for | 15 (94) | 25 (93) |
|  | Median FU (Range) | 7 (1-19) | 10 (1-79) |

*rHGG: CIR

^+^rHGG: in relation to primary tumor

**Supplementary Table 4:** Comparison of MRIvol evaluated pGBM/rHGG subcohorts and remaining patients for SUV max. T-test (two-sided) p-values.

| *pGBM (n=43)* | N=16 MRIvol | N=27 remaining | p-value |
| --- | --- | --- | --- |
| SUVmax (median, mad) | 3.84 (1.28) | 3.3 (1.30) | 0.28 |
| *rHGG (n=33)* | N=27 MRIvol | N=6 remaining |  |
| SUVmax (median, mad) | 2.97 (1.22) | 4.79 (3.01) | 0.13 |
